# Tuberculose neuro-méningée: profil clinique, paraclinique, thérapeutique et évolutif de 21 cas

**DOI:** 10.11604/pamj.2022.43.214.18038

**Published:** 2022-12-30

**Authors:** Abdellah Taous, Maha Ait Berri, Tarik Boulahri, Imane Traibi, Hicham Naji Amrani, Abdelhadi Rouimi

**Affiliations:** 1 Service de Neurologie, Hôpital Militaire Moulay Ismail, Meknès, Maroc,; 2 Service de Pneumologie, Hôpital Militaire Moulay Ismail, Meknès, Maroc

**Keywords:** Méningo-encéphalite, tuberculose, réaction polymérase en chaine, meningoencephalitis, tuberculosis, polymerase chain reaction

## Abstract

Les objectifs de cette étude étaient d'analyser les aspects épidémiologiques, cliniques, para-cliniques, thérapeutiques et évolutifs des patients atteints de la tuberculose neuroméningée. Il s'agit d'une étude rétrospective portant sur 21 patients hospitalisés entre janvier 2002 et décembre 2016 dans le service de neurologie pour une tuberculose neuroméningée. Les femmes étaient légèrement plus représentées que les hommes (SR=0,9), la tranche d´âge entre 20 et 40 ans était prédominante (47,61%). Le délai moyen du diagnostic était de 25 jours. Les symptômes inauguraux étaient principalement des signes généraux (100%). Les signes neurologiques étaient sous forme de céphalées (61,90%), de vomissements (47,61%) et un déficit moteur (33,33%). La ponction lombaire et la tomodensitométrie (TDM) cérébrale ont été réalisées dans 100% des cas. Tous les patients ont bénéficié d´un traitement anti-bacillaire et d´une corticothérapie. L´évolution était favorable dans 61,90% des cas, fatale dans 9,52% des cas et marquée par la persistance de séquelles neurologiques dans 28,57% des cas. La tuberculose neuro-méningée est une pathologie extrêmement polymorphe dans sa présentation clinique et radiologique. L´évolution est le plus souvent favorable si le diagnostic et la prise en charge sont précoces.

## Abstract (en)

The purpose of this study was to investigate the epidemiological, clinical, paraclinical, therapeutic and evolutionary profile of patients with neuromeningeal tuberculosis. We conducted a retrospective study of 21 patients with neuromeningeal tuberculosis hospitalized in the Neurological Department between January 2002 and December 2016. Women were slightly more represented than men (SR=0.9), the age group between 20 and 40 years (47.61%) was predominant. The mean time between symptom onset and diagnosis was 25 days. Inaugural symptoms mainly included common signs (100%). Neurological signs included headache (61.90%), vomiting (47.61%) and motor deficit (33.33%). Lumbar puncture and brain CT scan were performed in 100% of cases. All patients received antibiotic therapy and corticosteroids. Outcome was favorable in 61.90% of cases, fatal in 9.52% of cases and marked by the persistence of neurological sequelae in 28.57% of cases. Neuromeningeal tuberculosis is an extremely polymorphic disease in its clinical and radiological manifestation. Early diagnosis and management is mainly associated with a favorable outcome.

## Introduction

La tuberculose neuro-méningée (TNM) représente 5 à 15% des localisations extra-pulmonaires et constitue la forme la plus grave de l´infection par *Mycobacterium tuberculosis*. Elle est responsable de décès et de séquelles neurologiques graves dans plus de 50 % des cas malgré un traitement anti-bacillaire [1,2]. Ce pronostic est étroitement lié à la précocité du diagnostic et la qualité de la prise en charge [1]. Le grand polymorphisme clinique et le manque de spécificité des signes radiologiques et biologiques hormis l´identification du bacille de Koch (BK) par la réaction polymérase en chaine (PCR) au niveau du liquide céphalo-rachidien (LCR), rendent le diagnostic difficile et sont fréquemment responsables d´un retard dans la prise en charge. Les objectifs de l´étude étaient de décrire les aspects cliniques, paracliniques, thérapeutiques et évolutifs de l´ensemble de cas de TNM colligés au service.

## Méthodes

**Conception et contexte de l´étude:** il s´agit d´une étude rétrospective, qui a été conduite au service de neurologie de l´hôpital militaire Moulay Ismail de Meknès au Maroc, sur une période de 15 ans, entre janvier 2002 et décembre 2016.

**Population d´étude:** l´étude rapporte tous les dossiers classés TNM dans les registres d´hospitalisation. Seuls ont été retenus les dossiers répondant à nos critères d´inclusion. Au moins deux des critères suivants: un syndrome clinique évocateur méningé ou confusionnel fébrile et subaigu. La présence d´une méningite lymphocytaire à la ponction lombaire, une imagerie cérébrale évocatrice de lésions tuberculeuses, l´identification du BK par PCR ou à l´examen direct et la confirmation histologique après biopsie cérébrale.

**Collecte des données:** les sources des différentes données recueillies sur les fiches d´exploitation étaient les observations cliniques, les lettres des médecins généralistes ou spécialistes, les résultats des explorations paracliniques et le suivi noté par les médecins du service sur le dossier médical lors des consultations à titre externe.

**Méthodes statistiques:** l´analyse des données a été faite sur Excel 2010.

**Ethique:** les auteurs ont reçu l´accord de l´administration et du comité d´éthique.

## Résultats

**Caractéristiques générales:** l´âge moyen au moment du diagnostic était de 49,5 ans avec des extrêmes allant de 17 à 82 ans. La tranche d´âge la plus représentée au sein de la série est celle des 20 à 40 ans avec 47,61%, suivie de la tranche d´âge de 40 à 60 ans avec 28,57%. Le groupe des patients âgés de moins de 20 ans représentait 4,76% des cas et celui des patients âgés de plus de 60 ans, 19,04 % des cas. La médiane du délai du diagnostic entre le premier signe révélateur et le diagnostic de la maladie (date de la première PL) était de 25 jours environ. On a noté une légère prédominance féminine avec un sex-ratio de 0,9.

**Figure 1:**
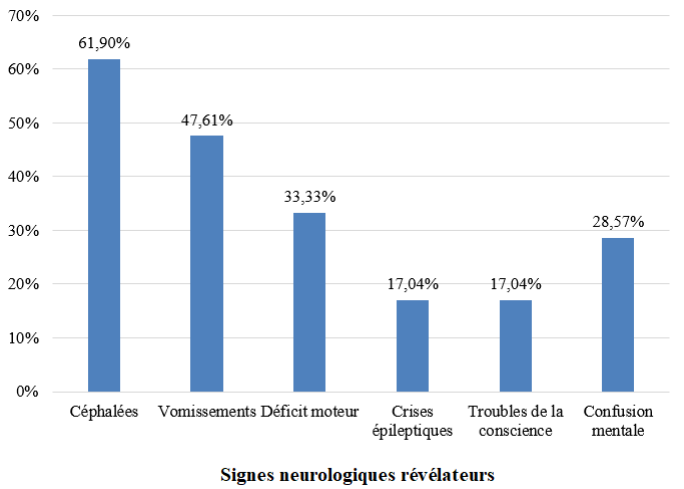
signes neurologiques révélateurs

**Figure 2:**
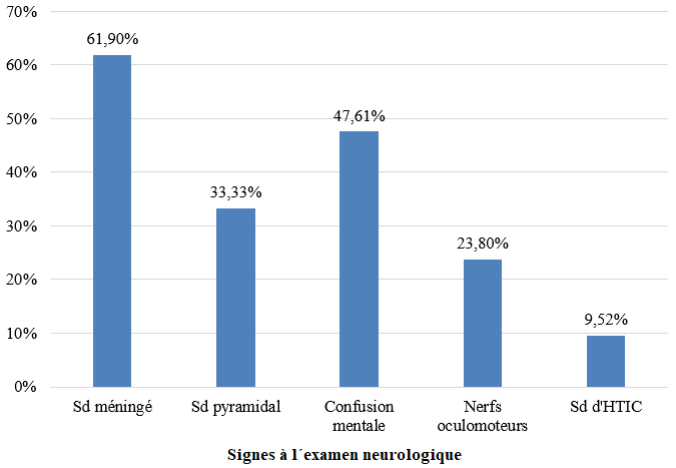
signes à l'examen neurologique

**Figure 3:**
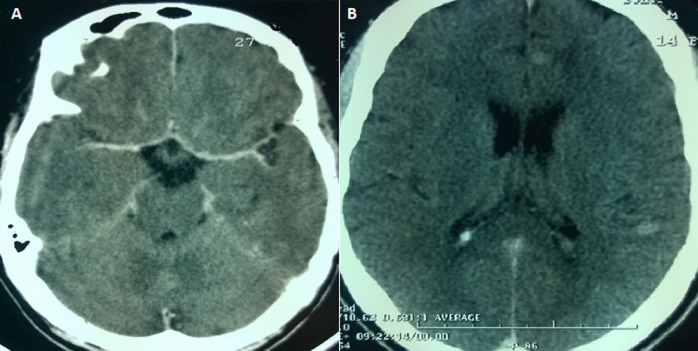
A) coupe axiale d'une TDM cérébrale objectivant une leptoméningite; B) coupe axiale d'une TDM cérébrale objectivant des tuberculomes hémisphériques gauches

**Figure 4:**
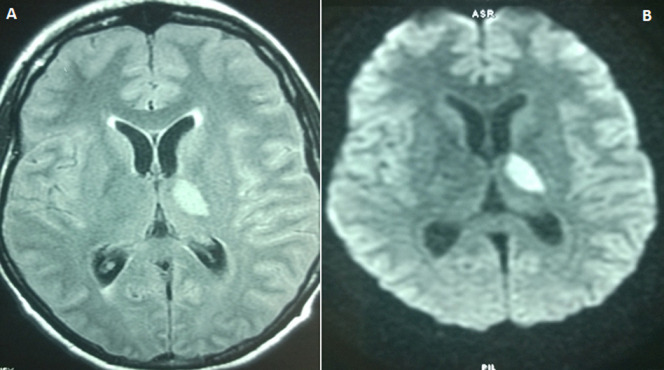
A) coupe axiale d'une IRM cérébrale en séquence flair objectivant un infarctus sylvien profond gauche; B) coupe axiale d'une IRM cérébrale en séquence diffusion objectivant un infarctus sylvien profond gauche

**Présentations clinique et paraclinique:** le mode d´installation de la maladie était subaigu dans 71,4% des cas et progressif dans 28,6% des cas. Les signes fonctionnels révélateurs et les signes de l´examen clinique sont résumés respectivement dans les figure 1, figure 2 et tableau 1. Tous les patients ont bénéficié d´une radiographie thoracique. Elle était interprétée comme pathologique chez 6 patients (28,5%) avec des lésions évocatrices de miliaire tuberculeuse chez 5 patients (23,8%) et des séquelles d´une tuberculose pulmonaire chez un patient (4,76%). Elle était normale chez 15 patients (71,5%). Tous nos patients ont bénéficié d´une recherche de BK dans les crachats, par tubage gastrique et dans les urines. Cette recherche était négative dans tous les cas. La TDM cérébrale a été réalisée chez 19 cas (90%). Elle s´est avérée normale chez 5 patients (23,81%), et a mis en évidence des lésions évocatrices de tuberculose neuro-méningée chez 14 cas (66,66%). Les signes scannographiques sont dominés par les signes de leptoméningite et les tuberculomes (Figure 3). Dix patients ont bénéficié d´une Imagerie par réRM cérébrale, elle était pathologique chez 7 d´entre eux. Les tuberculomes constituaient l´anomalie la plus fréquente (43%). Une image d´abcès cérébraux multiples a été notée chez un patient, et un infarctus sylvien profond chez deux patientes (Figure 4). L´imagerie par résonnance magnétique (IRM) médullaire a été réalisée chez 3 patients, elle a mis en évidence des images d´arachnoïdite spinale dans deux cas alors qu´elle était normale dans un cas. Tous les patients ont bénéficié d´une ponction lombaire initialement. L´analyse du LCR était anormale dans 20 cas sur 21. Les résultats de cette dernière sont détaillés dans le tableau 2.

**Tableau 1:** regroupement symptomatique

Forme clinique	Nombre de cas
Méningite basilaire	7 patients (33,33%)
Méningo-encéphalite	12 patients (57,14%)
Radiculo-myélite	01 patient (4,76%)
Méningo-encéphalo-radiculo-myélite	01 patient (4,76%)

**Tableau 2:** résultats de l´analyse du LCR

Analyse du LCR	Résultats (cas)
Aspect macroscopique	Clair dans 95% (20 cas)
Cytologie	≥ 5 GB/mm^3^: 95% (20 cas)
	≤ 05 GB/mm^3^: 4,8% (1 cas)
Formule	Prédominance lymphocytaire: 90% (18 cas)
	Prédominance PNN: 10% (2 cas)
Protéinorachie	≥ 0,3g/l: 10% (2 cas)
	≤ 0,3g/l: 20% (4 cas)
	≥ 1g/l: 70% (14 cas)
Glycorachie	≥ 0,5g/l: 14,3% (3 cas)
	≤ 0,5 g/l: 85,7% (18 cas)
Recherche de BK	Examen direct: positif dans un cas
	Culture: positive 1 cas
	PCR: positive 6 cas

**Traitement et résultats:** un traitement antituberculeux a été administré précocement chez tous nos patients. Le délai moyen de la mise en route de ce traitement était de cinq jours. Le traitement a fait appel à l´association de 4 anti-bacillaires RHZE (ERIP K4) dans 15 cas et RHZS dans 6 cas pendant 2 mois puis 2 anti-bacillaires RH pendant 7 à 9 mois. Une corticothérapie par voie générale à base de méthylprednisolone en IV au début puis un relais par voie orale par la prednisone a été administrée chez tous les patients. La dose moyenne de cette corticothérapie était de 50 mg/j et sa durée totale était de 3 mois avec une dégression progressive des doses à partir de la 6^e^semaine. Deux patients ont bénéficié d´un traitement neurochirurgical, il s´agissait d´une dérivation ventriculo-péritonéale d´une hydrocéphalie et d´un drainage chirurgical d´un abcès cérébral. L´évolution était favorable chez 13 patients, soit dans 61,90% et fatale chez 2 patients, soit 9,52% des cas. Six patients, soit 28,57% des cas ont gardé des séquelles neurologiques.

## Discussion

La tuberculose est une pathologie infectieuse grave, avec une incidence de 8 millions de nouveaux cas et un million de décès par an [1]. La TNM représente 10 à 30% des lésions expansives intracrâniennes dans les pays en voie de développement [3]. Elle est rare dans les pays industrialisés, mais de plus en plus fréquente depuis la pandémie du virus de l´immunodéficience humaine (VIH). Deux (2) à 5% des patients atteints d´une tuberculose ont une localisation cérébro-méningée associée, et la fréquence s´élève à 10% chez les patients séropositifs pour le VIH [3,4]. La TNM touche surtout le sujet jeune avec une moyenne d´âge variant entre 25 et 45 ans [4,5]. Dans notre série, l´âge moyen au moment du diagnostic était de 49,5 ans avec des extrêmes allant de 17 à 82 ans, et une médiane du délai de diagnostic de 25 jours. La répartition de la TNM en fonction du sexe est différemment appréciée dans la littérature. Pour la majorité des auteurs elle est à prédominance masculine.

Dans notre étude, le sex ratio est de 0,9. La forme clinique la plus fréquente au moment du diagnostic était la méningo-encéphalite, elle représentait 57,14% des cas, suivie de la méningite basilaire qui représentait 33,33% des cas et d´un cas de radiculo-myélite et un cas de méningo-encéphalo-radiculo-myélite. Ces résultats concordent globalement avec ceux des autres séries de la littérature [6-9]. Les images scannographiques étaient représentées par la prise de contraste méningée, les tuberculomes, l´hydrocéphalie, les lésions ischémiques et les abcès. La prise de contraste méningée était l´anomalie radiologique la plus fréquente (42,9%). Cette fréquence est supérieure à celle rapportée dans la littérature, 22% pour Bakhella *et al*., 23% pour Chelaïfa *et al*., 20,8% pour Marx *et al*. et 20% pour Gunawardhana *et al*. [6,7,10,11]. Les tuberculomes occupent la seconde place dans notre étude, soit 28,6% des cas. Cette fréquence rejoint celle de la série de Gunawardhana *et al*. [11].

L´IRM est plus sensible que la TDM dans la détection des tuberculomes de petite taille et ceux localisés au niveau du tronc cérébral [12], du cervelet et de la moelle épinière [13]. En effet, l´IRM a identifié des lésions de tuberculomes dans 42,8% des cas, tandis que la TDM était révélatrice de tuberculomes uniquement dans 28,6% des cas. Dans notre étude la culture était positive chez un seul patient soit (4,8%). Ce résultat est inférieur à celui rapporté dans la littérature (10 à 30%) [14-16]. Plus récemment le diagnostic de la tuberculose neuro-méningée (TNM) a bénéficié de l´apport des techniques d´amplification génique. En effet la PCR permet un diagnostic précoce et rapide dans les 24 heures [17]. Sa sensibilité est autour de 60%, alors que la spécificité avoisine 90% [18]. Dans notre série, la PCR a été réalisée chez 9 patients, elle était positive chez 6 d´entre eux, soit 66,67% des cas. Ce résultat est inférieur à celui de Ouhabi *et al*. qui avaient trouvé douze cas sur treize soit 92% [19]. Le traitement antituberculeux standard associe les quatre antituberculeux majeurs que sont H, R, E et Z pendant 2 mois, puis H et R pendant 7 mois selon les recommandations de la Société Française de Pneumologie [20] et pendant 7 à 10 mois pour le Conseil supérieur d´hygiène publique de France [21] et l´American Thoracic Society [22]. Nos patients ont été traités selon le schéma du ministère de la santé publique marocaine: 2RHZE (ERIP K4) dans 10 cas et RHZS dans 6 cas pendant 2 mois puis 2 antibacillaires RH pendant 7 à 9 mois. La corticothérapie, comme traitement adjuvant de la TNM, a été évaluée depuis plusieurs décennies [23].

La British Infection Society recommande, en s´appuyant sur plusieurs études et méta-analyses, l´instauration d´une corticothérapie pour tous les patients présentant une méningite tuberculeuse à la dose de 0,4mg/Kg/j, avec une décroissance des doses à partir de 6 à 8 semaines. La posologie prescrite et la durée avant décroissance des doses diffèrent pour le Royal College of Physicians (RCP) et le National Health Service (NHS). Le premier préconise une décroissance des doses de corticoïdes après 2 à 4 semaines de traitement à pleines doses, tandis que le second recommande la décroissance dans les 2 à 3 semaines suivant le début de la corticothérapie [24]. Dans notre série, tous les patients ont bénéficié d´une corticothérapie par voie générale à base de méthylprednisolone avec relais par voie orale par de la prednisone à la dose de 0,5 à 1 mg/Kg/j. La corticothérapie trouverait aussi sa place au côté des antibiotiques antituberculeux dans certaines circonstances. Elle diminue la mortalité et les séquelles neurologiques chez les patients présentant un tableau de gravité moyenne (confusion, signes de focalisation), d´autres auteurs montrent un bénéfice dans les cas graves comme le coma. Enfin, la corticothérapie diminuerait aussi le volume d´une hydrocéphalie [25]. Le pronostic de la TNM est dans l´ensemble, assez sévère, la mortalité est évaluée entre 15 et 40% [26]. Dans notre étude la TNM était responsable de 9,52% de décès, résultat qui reste inférieur à celui de Dollo *et al*. [8] qui avaient trouvé 31%.

## Conclusion

La TNM demeure une infection sévère très polymorphe pouvant engager le pronostic vital. Le diagnostic a longuement bénéficié de l´apport de l´imagerie médicale, notamment la TDM et l´IRM. Sa confirmation bactériologique n´est pas toujours aisée et lorsqu´elle existe, elle est souvent tardive. Seul un diagnostic précoce et un traitement initié suffisamment tôt sont garants d´un bon pronostic. Enfin, la prise en charge des patients pourrait être améliorée par l´adoption d´un consensus de diagnostic universel reposant sur des critères diagnostiques standardisés.

### Etat des connaissances sur le sujet

- *La tuberculose neuroméningée est une pathologie à présentation clinique et radiologique extrêmement polymorphe;*
- *Elle est responsable de décès et de séquelles neurologiques graves dans plus de 50% des cas malgré un traitement anti-bacillaire;*
- *Son pronostic est étroitement lié à la précocité du diagnostic et la qualité de la prise en charge thérapeutique.*

### Contribution de notre étude à la connaissance

- *Notre travail reflète ce grand polymorphisme clinique et illustre bien les variétés de localisation de la tuberculose neuroméningée;*
- *Notre travail souligne l´apport considérable de l´imagerie dans le diagnostic précoce de la tuberculose neuroméningée notamment l´IRM cérébrale;*
- *A la lumière des résultats de notre travail, nous soulevons l´importance d´un consensus de diagnostic universel reposant sur des critères diagnostiques standardisés.*
